# Vaccination and Beauty

**Published:** 1899-02

**Authors:** 


### Vaccination and Beauty.

Two interesting letters have recently been published giving some reminiscences of what smallpox was before vaccination became general or compulsory. The Duke of Argyle writes to deplore the mania which has set in against vaccination, and attributes it to a "long immunity from disease, which has led to a discreditable and stupid forgetfulness of its cause. The highlands of Scotland in the last century used to be swept by the pestilence to a fearful extent. 'Decimation' is now inadequate to convey an idea of the effect on the population. Whole parishes were depopulated. All this stopped when inoculation came, and vaccination subsequently. No fact in history is more clearly established." The other letter is from Mr. Holman Hunt, R.A., who says that about the year 1862 he asked the veteran judge, the Right Honorable Stephen Lushington, what was his view of the relative beauty of ladies "seventy years ago" and in the passing day. Mr. Hunt said he asked the question not without some reserved recognition of the prejudice that lies ever in aged breasts for the glories of the days of youth. But the answer he got from Dr. Lushington astonished him. "Well," he exclaimed, "you can have no idea how it was that not more than one person in twenty was then undisfigured by traces of the smallpox, and this generally to such a degree that whatever beauty there had been was very seriously marred. It followed then that when a woman had escaped there was a disposition to regard her as a beauty for this cause alone, and if with this she had chiseled, symmetrical features and good form of face and figure, too, the admiration of her amounted to worship. The difference I see now is that every lady we meet would have been a beauty in my early days."—*British Journal of Dental Science.*

